# Identifying risk factors of recurrence for clinically node negative papillary thyroid carcinoma with pathologic N1a

**DOI:** 10.1186/s12893-019-0541-5

**Published:** 2019-07-05

**Authors:** Young Jae Ryu, Jin Seong Cho, Min Ho Park, Jung Han Yoon

**Affiliations:** Department of Surgery, Chonnam National University Medical School, 42 Jebong-ro, Dong-gu, Gwangju, 61469 Republic of Korea

**Keywords:** Papillary thyroid cancer, Prophylactic central neck dissection, Recurrence

## Abstract

**Background:**

Whether or not to perform prophylactic central lymph node dissection (CLND) in the case of clinically node-negative papillary thyroid cancer (PTC) is controversial. The purpose of this study was to investigate the risk factors for recurrence in clinically node-negative PTC patients who underwent total thyroidectomy plus bilateral central neck dissection and was verified pathologic N1a.

**Methods:**

We retrospectively reviewed the medical records of 1082 PTC patients who underwent total thyroidectomy and prophylactic bilateral CLND between January 2004 and December 2012. We used Cox-proportional hazard regression analyses in order to explore potential predictive factors for recurrence.

**Results:**

During a median follow-up (range) of 78 (12–158) months, recurrence occurred in 62 (5.7%) patients. Main tumor size more than 1 cm, gross extrathyroidal extension (ETE), positive lymph node (LN) more than 3, and LN ratio > 0.5 were all significantly associated with recurrence according to univariate analysis. In model I multivariate analysis (tumor size, gross ETE, LN ratio), LN ratio > 5 (hazards ratio [HR], 4.794; 95% confidence interval [CI], 2.674–8.595; *p* < 0.001) was found to be predictive of recurrence. Gross ETE (HR, 1.794; 95% CI, 1.024–3.143; *p* = 0.041) and positive LN more than 3 (HR, 2.505; 95% CI, 1.513–4.146; *p* < 0.001) were predictors for recurrence in model II multivariate analysis (tumor size, gross ETE, the number of positive LN).

**Conclusions:**

We recommend that surgeons try to focus completely on performing prophylactic CLND for patients with suspicious gross ETE during preoperative evaluation. Close monitoring and thorough management are needed for clinically node-negative PTC patients with LN ratio of more than 0.5 and more than 3 positive LN in the central compartment.

## Background

Papillary thyroid cancer (PTC) is the most common histologic type of thyroid cancer. Although the involvement of lymph node (LN) in PTC is high upon the initial diagnosis due to the lymphogenous spread pattern, PTC patients have better survival outcomes than patients with other thyroid malignancies, with an over 95% survival rate in 10 years and 93% survival in 20 years [1]. On the other hand, the recurrence rate during follow-up can be up to 30% in PTC [2, 3]. One study reported that up to 90% of PTC patients with recurrence had LN metastasis at first operation, and that the presence of LN metastases is associated with mortality due to thyroid cancer [4]. The detection of LN metastasis prior to surgery is somewhat difficult. The diagnosis of micro PTC that is below 1 cm in tumor size has become easier through the introduction of high resolution ultrasonography (US). From the perspective of detecting LN, distinguishing suspicious LN in the central compartment is harder than in the lateral compartment. Besides, the sensitivity of US and computed tomography (CT) is lower for detecting LN in the central compartment because of the anatomical location (proximity to the thyroid and trachea) [5].

Therapeutic central LN dissection (CLND) using the same incision is acceptable for the management of clinically node-positive PTC; however, performing prophylactic CLND for clinically node-negative PTC remains controversial. According to recent guidelines from the American Thyroid Association (ATA), prophylactic CLND should be considered in patients with clinically central node-negative PTC who have advanced primary tumors (T3 or T4) or who are clinically node-positive in the lateral compartment, but it is not recommended for patients with small (T1 or T2), noninvasive, or clinically node-negative PTC [6]. In addition to the ATA guidelines, the European Society of Endocrine Surgeons has suggested that prophylactic CLND is appropriate for patients aged 45 years and older or 15 years and younger, male gender, and patients with bilateral or multifocal tumors [7]. There is still a lack of evidence showing that the removal of as much LN as possible gives rise to better survival outcomes. The ultimate goal of prophylactic CLND is not the improvement of survival, but the reduction of reoperation that might cause higher rates of complications as well as the facilitation of postoperative follow-up using thyroglobulin (Tg). In addition, prophylactic CLND is helpful for achieving accurate nodal stage and decision-making of radioactive iodine (RAI) therapy. Even when LN metastases are revealed, clinicians can consider the omission of RAI therapy for patients with lower risk of recurrence in PTC.

It is generally accepted that the extending pattern of LNs in PTC begins from the central compartment and progresses toward the lateral compartment. The patients who underwent prophylactic CLND had subclinical LN metastasis up to 50% [8, 9]. Several studies have focused on the efficacy of prophylactic CLND; however, there have been few studies on PTC patients who underwent prophylactic CLND with confirmed pathologic N1a. Hence, the aim of this study was to investigate the risk factors for recurrence in clinically node-negative PTC patients who underwent total thyroidectomy plus bilateral central neck dissection with verified pathologic N1a.

## Methods

### Patients

We reviewed the medical records of 9135 patients who underwent thyroid surgery at Chonnam National University Hwasun Hospital between January 2004 and December 2012. Exclusion criteria were as followings: patients who were under 15 years old, who underwent completion thyroidectomy due to recurrence of any histologic type, who underwent thyroidectomy due to benign thyroid disease or other thyroid malignancy besides PTC, who underwent lateral neck dissection at first surgery, who were clinically node-positive in the central and lateral compartment, who did not undergo CLND, who were confirmed as having pathologic N0, who did not achieve R0 resection, who had distant metastasis upon first diagnosis, who had abnormal thyroid function test results prior to operation, who had less than a one year follow-up period, who experienced recurrence within one year after surgery, and who had other malignancies pre- or postoperatively. We enrolled a total of 1082 patients who underwent total thyroidectomy and prophylactic bilateral CLND in this study. All patients were carefully reviewed preoperative neck US and neck CT for the evaluation of the LNs in the central neck compartment. This retrospective study was approved by our hospital’s institutional review board and permission is required to access the data.

All patients were confirmed as having Bethesda category V (154 patients, 14.2%) or VI (928 patients, 85.8%) PTC through preoperative fine needle aspiration cytology (FNAC). We exhaustively collected patients’ clinicopathological information, LN ratio (number of metastatic LNs divided by number of harvested LNs), complications related to operation, and recurrence. Cancer staging followed the recent recommendations outlined by American Joint Committee on Cancer (AJCC) [10].

### Operation and postoperative follow-up

All patients underwent total thyroidectomy and comprehensive bilateral CLND in level VI (Delphian, pre- and paratracheal, and/or paraesophageal node) and level VII (upper mediastinal node). All surgeries were performed by the same thyroid surgical team. Specimens were sent to the pathologic department and meticulously examined by experienced pathologists. Based on the tumor and node characteristics, patients received 30 − 150 mCi of RAI therapy at two to three months following surgery. However, the omission of RAI therapy was considered in some cases because of the clinician’s decision or patient’s refusal. The interval of follow-up was three to six months for the first three years and thereafter annual. All patients received regular physical examinations, neck US, chest radiography, measurement of serum-free thyroxine and thyrotropin, Tg, and anti-Tg antibody concentrations every visit. If necessary, neck CT, whole-body iodine scanning, and 18F-fluorodeoxyglucose positron emission tomography CT were performed for further evaluation. We defined recurrence as structural recurrence, which was evaluated using image modalities and histologic examinations. Suspicious structural recurrence was confirmed with FNAC, if accessible. Reoperation followed by RAI therapy was applicable to most patients with structural recurrence; however, if the lesion of recurrence was unresectable distant organ, RAI therapy was considered as the best treatment option rather than reoperation.

### Complications

We determined wound infections to be cases requiring local treatment including antibiotics because of cellulitis or those needing incision and drainage because of deep neck infection. The time of distinction between transience and permanence, which is applicable to hypoparathyroidism and recurrent laryngeal nerve injury, was six months after the initial surgery. According to our institution’s protocols, the measurement of parathyroid hormone (PTH) with the level of total calcium and ionized calcium was examined in six hours, one day, and two days postoperatively for patients who underwent thyroid surgery. We defined permanent hypoparathyroidism as below-normal serum PTH with low level of calcium that should be maintained calcium and vitamin D supplementation more than 6 months after surgery. Unfortunately, the patients enrolled in this study did not routinely undergo preoperative flexible laryngoscopy. However, selective flexible laryngoscopy was performed for patients who had suspicious gross ETE towards tracheoesophageal groove or symptoms related to voice changes preoperatively, who developed voice changes or had suspicious of recurrent laryngeal nerve injury postoperatively. We defined postoperative bleeding to be cases requiring conservative treatment or reoperation.

### Statistical analysis

The primary end point was any structural recurrence in the loco-region or distant organs. Recurrence-free survival (RFS) was defined as the period between the first operation and the detection of recurrence. Continuous variables are presented as median (range) or mean (standard deviation); categorical variables are presented as number (percent). We used a univariate Cox-proportional hazards model in order to analyze the relationships between the clinicopathological variables and RFS. We also conducted multivariate Cox-proportional hazards regression analyses by means of backward elimination using the variables with *p* < 0.05 in the univariate analyses. The receiver operating characteristic curve was used to calculate an optimal LN ratio cutoff. The Kaplan-Meier curve with log-rank test was utilized for comparison of RFS according to LN ratio, number of metastatic LN, and ETE. All statistical analyses were performed using SPSS version 23.0 (IBM Inc., Armonk, NY, USA) and defined statistical significance to be p less than 0.05.

## Results

### Patients’ demographics

Of the total 1082 patients, 213 (19.7%) were male. The median (range) age was 46 years (15–75), and 260 patients (24.0%) were over 55. The mean primary tumor size was 1.15 cm (standard deviation, 0.74 cm), and 430 patients (39.7%) had a main tumor more than 1 cm in size, while 652 (60.3%) patients had microcarcinoma. There were 906 patients (83.7%) with tumors limited to the thyroid (T1, T2, T3a), 119 (11.0%) with tumors with gross ETE invading only strap muscles (T3b), and 57 (5.3%) with stage T4a tumors. Six hundred thirty-five (58.7%) patients had more than five harvested central lymph nodes. Three hundred thirty-one (30.6%) patients and 242 (22.4%) patients had tumor multifocality and bilaterality, respectively. We observed chronic lymphocytic thyroiditis in 334 (30.9%) patients and lymphovascular invasion in 23 (2.1%) patients. Nine hundred eighty-six (91.1%) patients were administered postoperative RAI therapy. According to AJCC TNM stage, 950 (87.7%) patients belonged to stage I; 116 (10.7%), stage II; and 16 (1.5%), stage III. The median follow−up (range) was 78 (12–158) months (Table 1).

**Table 1:** Patients’ demographics

Variables	Number (percent)
Age^§^	46.0 years (15–75)
< 55 years	822 (76.0)
≥ 55 years	260 (24.0)
Sex	
Female	869 (80.3)
Male	213 (19.7)
Underlying disease	
Hypertension	183 (16.9)
Diabetes	71 (6.6)
Tumor size^*^	1.15 cm ± 0.74
≤ 1 cm	652 (60.3)
> 1 cm	430 (39.7)
T stage	
T1a	622 (57.5)
T1b	230 (21.3)
T2	51 (4.7)
T3a	3 (0.3)
T3b	119 (11.0)
T4a	57 (5.3)
Number of harvested lymph node	
≤ 4	447 (41.3)
≥ 5	635 (58.7)
Multifocality	331 (30.6)
Bilaterality	242 (22.4)
Chronic lymphocytic thyroiditis	334 (30.9)
Lymphovascular invasion	23 (2.1)
TNM stage	
Stage I	950 (87.8)
Stage II	116 (10.7)
Stage III	16 (1.5)
Radioactive iodine therapy	986 (91.1)
Recurrence	62 (5.7)
follow-up^§^	78 months (12–158)
Total patients	1082 (100)

§ represents median and range, * represents mean and standard deviation

During follow-up, recurrence occurred in 62 (5.7%) patients. The mean time to recurrence was 36 months (range, 12–102 months). Of these 62 patients, 19 only had recurrence in the operative bed (15 patients) or central neck compartment (four patients), while 32 only had it in the lateral neck compartment. Nine patients had recurrence in the central and lateral compartments simultaneously. Two patients had distant metastasis confined to the lung.

### Univariate analyses according to recurrence

**Fig. 1:**
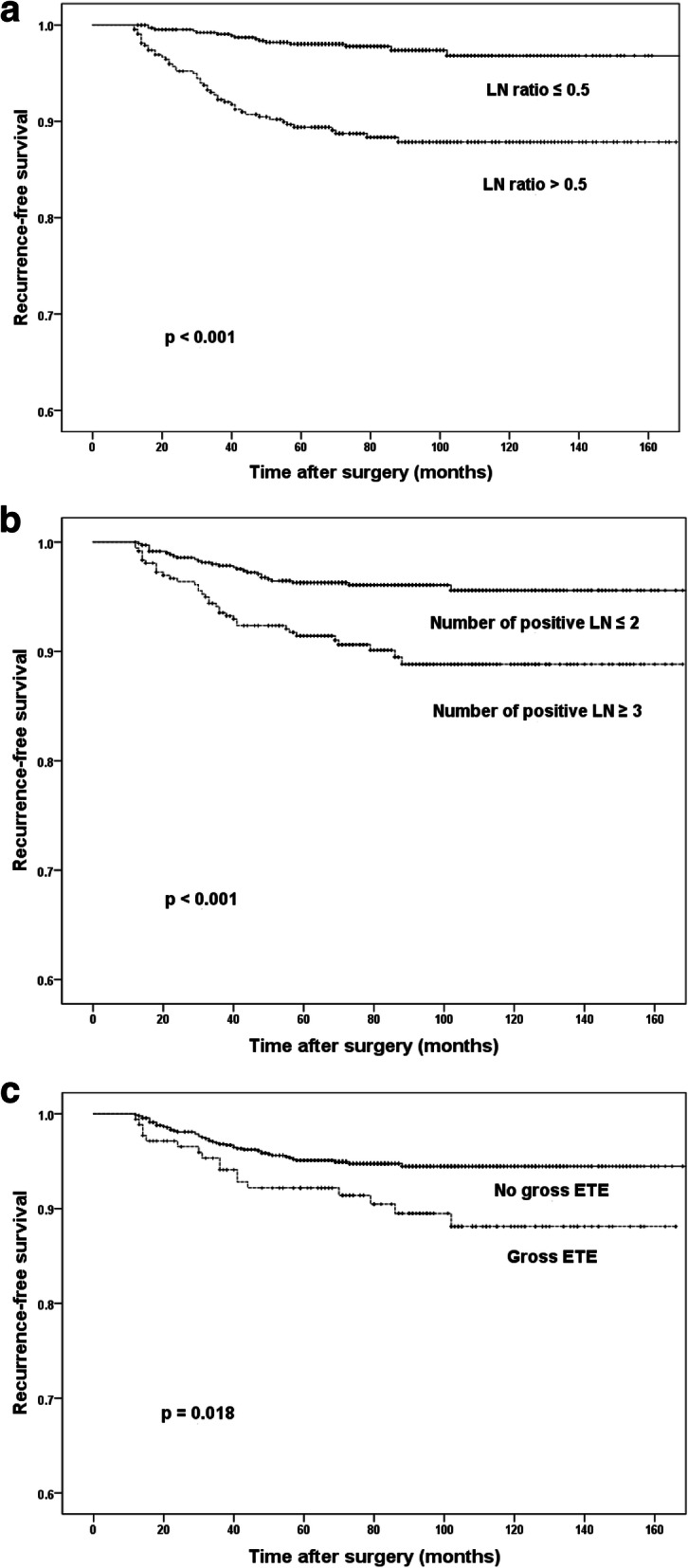
Kaplan-Meier curves according to variables. LN ratio (a), Number of positive LN (b), Gross ETE (c). ETE = Extrathyroidal extension, LN = Lymph node

Univariate analysis using the Cox-proportional hazards regression model for all patients showed that main tumor size more than 1 cm (vs. ≤ 1 cm; *p* = 0.047), gross ETE (vs. no gross ETE; *p* = 0.018), positive LN more than 3 (vs. ≤ 2; *p* < 0.001), and LN ratio > 0.5 (vs. ≤ 0.5; *p* < 0.001) were all significantly associated with recurrence. There were no significant effects of age, gender, multifocality, bilaterality, LVI, CLT, number of harvested LNs, or TNM stage (Table 2, Fig. 1).

**Table 2:** Univariate Cox Regression of recurrence

Variables	HR	95% CI	*p*
Age			
< 55 years	1		
≥ 55 years	0.674	0.351–1.294	0.236
Sex			
Female	1		
Male	1.258	0.694–2.282	0.450
Tumor size			
≤ 1 cm	1		
> 1 cm	1.656	1.006–2.725	0.047
Multifocality			
No	1		
Yes	1.543	0.929–2.562	0.094
Bilaterality			
No	1		
Yes	1.115	0.624–1.995	0.713
LVI			
No	1		
Yes	0.891	0.515–1.543	0.891
CLT			
No	1		
Yes	0.891	0.515–1.543	0.681
T stage			
T1/2/3a	1		
T3b	1.013	0.432–2.376	0.976
T4a	4.019	2.079–7.773	< 0.001
ETE			
No gross ETE	1		
Gross ETE	1.964	1.124–3.433	0.018
Harvested LN			
≤ 4	1		
≥ 5	0.613	0.372–1.010	0.055
Positive LN			
≤ 2	1		
≥ 3	2.606	1.577–4.306	< 0.001
LN ratio			
≤ 0.5	1		
> 0.5	4.992	2.791–8.928	< 0.001
TNM stage			
Stage I	1		
Stage II/III	0.507	0.184–1.398	0.189

CI = Confidence interval, CLT = Chronic lymphocytic thyroiditis, ETE = Extrathyroidal extension, HR = Hazards ratio, LN = Lymph node, LVI = Lymphovascular invasion

### Multivariate analyses according to recurrence

We divided the number of positive LN and LN ratio to avoid the interference in nodal factors. Multivariate Cox-proportional hazards regression analyses revealed that LN ratio > 5 (hazards ratio [HR], 4.794; 95% confidence interval [CI], 2.674–8.595; *p* < 0.001) was a predictor of recurrence in model I (tumor size, gross ETE, LN ratio), and that gross ETE (HR, 1.794; 95% CI, 1.024–3.143; *p* = 0.041) and positive LN more than 3 (HR, 2.505; 95% CI, 1.513–4.146; *p* < 0.001) were predictors of recurrence in model II (tumor size, gross ETE, the number of positive LN) (Table 3).

**Table 3:** Multivariate Cox Regression of recurrence

Variables	Model I			Model II		
	HR	95% CI	*p*	HR	95% CI	*p*
Tumor size						
≤ 1 cm	1			1		
> 1 cm	1.260	0.754–2.105	0.377	1.354	0.808–2.268	0.249
ETE						
No gross ETE	1			1		
Gross ETE	1.645	0.939–2.883	0.082	1.794	1.024–3.143	0.041
LN ratio						
≤ 0.5	1					
> 0.5	4.794	2.674–8.595	< 0.001			
Positive LN						
≤ 2				1		
≥ 3				2.505	1.513–4.146	< 0.001

CI = Confidence interval, ETE = Extrathyroidal extension, HR = Hazards ratio, LN = Lymph node

### Complications

Of the total 1082 PTC patients, no patient suffered from wound infection. The incidence rates of transient and permanent hypoparathyroidism were 5.8% (63 patients) and 3.4% (37 patients), respectively; we observed transient and permanent recurrent laryngeal nerve injury in 32 (3.0%) and 10 (0.9%) patients. Two patients underwent reoperation due to postoperative bleeding (Table 4).

**Table 4:** Postoperative complications

Complication	Number (%)
Wound infection	0
Hypocalcemia	
Transient	63 (5.8)
Permanent	37 (3.4)
Recurrent laryngeal nerve injury	
Transient	32 (3.0)
Permanent	10 (0.9)
Bleeding	2 (0.2)

## Discussion

Through this study, we aimed to determine the predictors of recurrence in 1082 clinically node-negative PTC patients who underwent total thyroidectomy plus bilateral CLND and who had proven pathologic N1a. During a follow-up period of 78 months (range, 12–158), 62 patients experienced recurrence which was predicted by gross ETE and nodal factors (LN ratio > 0.5, the number of positive LN ≥ 3).

There are three main strategies regarding the management of thyroid cancer: surgery, thyroid stimulating hormone suppression, and RAI ablation therapy. Of these three modalities, clinicians think that surgical completeness is the most important. Complete resection of the LN is important for surgical completeness, along with removal of the primary tumor. It is understandable that patients who are clinically node-negative do not need prophylactic lateral neck dissection; however, prophylactic CLND is still a debatable issue in terms of management in clinically node-negative PTC patients.

Prophylactic CLND may have the possibility of upstaging in considerable number of patients with PTC and induce proper N stage for RAI therapy. Hughes et al. found that prophylactic CLND has resulted in upstaging for one-third patients older than 45 years and has also resulted in the modification of dose in RAI therapy [11]. Another study reported that 30% PTC patients with T1 tumor who underwent prophylactic CLND can be indicated for RAI therapy [12]. However, the patients with pathologic N0 following prophylactic CLND may have the advantage of low-dose RAI or no RAI therapy based on tumor features. Therefore, prophylactic CLND provides important information regarding more tailored management of RAI therapy and postoperative follow-up using Tg in PTC patients with subclinical LN metastases.

Meta-analysis reported that prophylactic CLND decrease locoregional recurrence and had the opposite relationship in terms of postoperative hypocalcemia [13, 14]. Another meta-analysis showed that the recurrence rate is 2.02% in patients who underwent thyroidectomy plus prophylactic CLND and 3.92% in patients who underwent only thyroidectomy; however, prophylactic CLND was not significantly associated with local recurrence (odds ratio = 1.05, 95% confidence interval 0.48–2.31) [15]. Wang et al. concluded that there were no significant differences in recurrence and long term complications between patients who underwent only total thyroidectomy and those who underwent total thyroidectomy plus prophylactic CLND [16]. Considering the optimal (maximal) outcomes and quality of life in clinically node-negative PTC patients, prophylactic CLND is an imperative topic to be considered by the thyroid surgeons. The rates of surgery-related permanent complications may be lower when not performing prophylactic CLND. When recurrence occurs, repeated thyroid surgery is more challenging due to the formation of scar tissue and distorted anatomy, which may lead to the increased risk of permanent complications. Recurrence in the central compartment for patients who did not undergo prophylactic CLND in the first operation is more difficult to identify than that in the lateral compartment [17]. The rates of complication are closely associated with the surgical extent and could be the reason for the curtailment of the surgical extent in PTC. Patients who undergo reoperation due to recurred PTC have a high incidence of several operations in the future and consequently do not achieve a biochemical complete response during follow-up [18, 19]. Reoperation in the central compartment by an experienced thyroid surgeon is a safe procedure and does not increase the risk of surgical complications compared to initial prophylactic CLND [20, 21]. Previous research has reported that prophylactic CLND is safe as performed by high-volume thyroid surgeon [22, 23]. Major development in technology, such as energy devices and intraoperative neuro-monitoring may lead to lower surgery-related complications, especially reoperation.

The factors that are associated with aggressive disease in PTC are age, male gender, large primary tumor size, and PTC variant histologic type such as tall cell, columnar cell, Hurthle cell, diffuse sclerosis, and insular variant. The features of the primary tumor are one of the salient factors for predicting LN involvement in the central compartment and helpful for deciding the extent of thyroidectomy [24]. ETE has been regarded as one of the important independent prognostic factors for persistent and recurrent PTC and has been slightly changed according to the most recent AJCC staging manual. The meaning of minor ETE was excluded because of equivalent survival outcomes between patients with minor ETE and those without ETE. In addition, tumor > 4 cm in the greatest dimension bounded to the thyroid gland is considered T3a and gross ETE with invasion only to the strap muscles is considered T3b [10]. This present study also recognized that there was no significant association according to recurrence among patients with or without minor ETE. Also, patients with gross ETE had a higher recurrence rate than those without ETE. Therefore, meticulous CLND is needed for clinically node-negative PTC patients with gross ETE during preoperative evaluation and intraoperative finding.

In the study with unilateral PTC and clinically node-negative patients who underwent total thyroidectomy plus bilateral CLND, authors reported 42.9% unilateral subclinical metastases in the central compartment and a high possibility of subclinical metastases if the tumor was more than 1 cm in size [25]. Provided that the distinction of LN involvement is possible prior to operation, surgeons can decide between therapeutic or prophylactic CLND depending on LN status; however, determining whether or not there is suspicious LN in the central compartment prior to surgery is very difficult. In addition, the sensitivity of preoperative US and CT is relatively low to detect suspicious LN in the central compartment [26]. Some authors have suggested that intraoperative frozen section examination of the ipsilateral central LN should be the primary surgical approach option taken prior performing bilateral CLND for clinically node-negative PTC [27]. Although the introduction of this approach is relatively short for the analysis of PTC patients in our institution, it may suitable for recent trends that are changing to minimize the extent of thyroid surgery. It is generally accepted that the lymphatic spread pattern of PTC begins in the central compartment at first, followed afterward by the lateral compartment. Using this concept, some surgeon have adopted sentinel LN biopsy, which is used for melanoma and breast cancer to decide the surgical extent of LN intra-operatively; however, there is still a point to be overcome before applying it to PTC because of its high false negative rate [28].

In the study with clinically node-negative PTC patients during a median follow-up of 164 months, the authors revealed that 59% of total patients were confirmed N1a on postoperative pathologic result [9]. They demonstrated that age (older than 55 years), tumor size (more than 2 cm), significant ETE, and the number of confirmed central LN metastases (more than 5) were associated with recurrence in the central compartment [9]. A considerable number of clinically node-negative PTC patients were confirmed as having pathologic N1a. Therefore, it is important to achieve complete removal of LN in the central compartment when performing the first thyroid surgery. Although there have been several studies regarding the efficacy of prophylactic CLND in PTC, most studies have included PTC patients with pathologic N1a and N0, and some research has classified clinically node-negative PTC patients who underwent only thyroidectomy as N0 stage [8, 29]. This present study excluded clinically node-negative PTC with pathologic N0, as we tried to find out the predictive factors of recurrence in clinically node-negative PTC with pathologic N1a. Although subclinical LN metastasis was observed in up to 50% of clinically node-negative PTC patients, nothing has yet been determined about the number of harvested central LN when performing CLND in thyroid surgery. We did not observe an optimal number of harvested central LN in this study. According to the recently published AJCC 8th edition, N0a is defined as one or more cytologically or histologically confirmed benign lymph nodes [10]. Although we performed comprehensive CLND, some patients had a small number of central LN on the pathologic results. In order to supplement this, we additionally analyzed LN ratio. This study revealed that the number of positive central LNs and the LN ratio could be important indicators supplementing the current TNM stage.

This study has several limitations. First, this study design is retrospective and some patients had relatively short-term follow-up period. Second, nine hundred eighty-six (91.1%) patients underwent RAI therapy in this study; however, many of those patients were classified as low risk group, RAI therapy can potentially be omitted in such cases according to recent guidelines; we did not consider the level of stimulated Tg or the influence of RAI therapy. Third, we did not consider biochemical incomplete response regarding the level of Tg and anti-Tg antibody during postoperative follow-up. In addition, certain patients showed undetectable levels of Tg and anti-Tg antibody, irrespective of having structural recurrence. Fourth, we did not include the statuses of involved LNs such as size and extranodal extension. We are planning to collect sufficient clinicopathological data on long-term follow-up results in PTC patients who underwent prophylactic CLND.

## Conclusions

Performing prophylactic CLND in clinically node-negative PTC patients is a useful and efficient procedure. We recommend that surgeons try to focus on complete performing prophylactic CLND for patients with suspicious gross ETE during preoperative evaluation. Close monitoring and thorough management are needed for clinically node-negative PTC patients with more than 0.5 of LN ratio and more than three positive LNs in the central compartment.

## Data Availability

The datasets used and analyzed during the current study available from the corresponding author on reasonable request.

## Abbreviations

AJCC: American Joint Committee on Cancer
ATA: American Thyroid Association
CI: Confidence interval
CLND: Central lymph node dissection
CLT: Chronic lymphocytic thyroiditis
CT: Computed tomography
ETE: Extrathyroidal extension
FNAC: Fine needle aspiration cytology
HR: Hazards ratio
LN: Lymph node
LVI: Lymphovascular invasion
PTC: Papillary thyroid cancer
PTH: Parathyroid hormone
RAI: Radioactive iodine
RFS: Recurrence-free survival
Tg: Thyroglobulin
US: Ultrasonography
